# Pioglitazone Inhibits Diabetes-Induced Atrial Mitochondrial Oxidative Stress and Improves Mitochondrial Biogenesis, Dynamics, and Function Through the PPAR-γ/PGC-1α Signaling Pathway

**DOI:** 10.3389/fphar.2021.658362

**Published:** 2021-06-14

**Authors:** Zhiwei Zhang, Xiaowei Zhang, Lei Meng, Mengqi Gong, Jian Li, Wen Shi, Jiuchun Qiu, Yajuan Yang, Jianping Zhao, Ya Suo, Xue Liang, Xinghua Wang, Gary Tse, Ning Jiang, Guangping Li, Yungang Zhao, Tong Liu

**Affiliations:** ^1^ Tianjin Key Laboratory of Ionic-Molecular Function of Cardiovascular Disease, Department of Cardiology, Tianjin Institute of Cardiology, Second Hospital of Tianjin Medical University, Tianjin, China; ^2^ Department of Gastroenterology, The First Affiliated Hospital of Xi’an Jiao Tong University, Shanxi, China; ^3^ Department of Cardiology, Tianjin Hospital, Tianjin, China; ^4^ Tianjin Key Laboratory of Exercise Physiology and Sports Medicine, Department of Health and Exercise Science, Tianjin University of Sport, Tianjin, China

**Keywords:** atrial fibrillation, diabetes mellitus, pioglitazone, mitochondria, oxidative stress

## Abstract

**Background:** Oxidative stress contributes to adverse atrial remodeling in diabetes mellitus. This remodeling can be prevented by the PPAR-γ agonist pioglitazone *via* its antioxidant and anti-inflammatory effects. In this study, we examined the molecular mechanisms underlying the protective effects of pioglitazone on atrial remodeling in a rabbit model of diabetes.

**Methods:** Rabbits were randomly divided into control, diabetic, and pioglitazone-treated diabetic groups. Echocardiographic, hemodynamic, and electrophysiological parameters were measured. Serum PPAR-γ levels, serum and tissue oxidative stress and inflammatory markers, mitochondrial morphology, reactive oxygen species (ROS) production rate, respiratory function, and mitochondrial membrane potential (MMP) levels were measured. Protein expression of the pro-fibrotic marker TGF-β1, the PPAR-γ coactivator-1α (PGC-1α), and the mitochondrial proteins (biogenesis-, fusion-, and fission-related proteins) was measured. HL-1 cells were transfected with PGC-1α small interfering RNA (siRNA) to determine the underlying mechanisms of pioglitazone improvement of mitochondrial function under oxidative stress.

**Results:** The diabetic group demonstrated a larger left atrial diameter and fibrosis area than the controls, which were associated with a higher incidence of inducible atrial fibrillation (AF). The lower serum PPAR-γ level was associated with lower PGC-1α and higher NF-κB and TGF-β1 expression. Lower mitochondrial biogenesis (PGC-1α, NRF1, and TFAM)-, fusion (Opa1 and Mfn1)-, and fission (Drp1)-related proteins were detected. Mitochondrial swelling, higher mitochondrial ROS, lower respiratory control rate, and lower MMP were observed. The pioglitazone group showed a reversal of structural remodeling and a lower incidence of inducible AF, which were associated with higher PPAR-γ and PGC-1α. The pioglitazone group had lower NF-κB and TGF-β1 expression levels, whereas biogenesis-, fusion-, and fission-related protein expression was higher. Further, mitochondrial structure and function were improved. In HL-1 cells, PGC-1α siRNA transfection blunted the effect of pioglitazone on Mn-SOD protein expression and MMP collapse in H_2_O_2_-treated cells.

**Conclusion:** Diabetes mellitus induces adverse atrial structural, electrophysiological remodeling, and mitochondrial damage and dysfunction. Pioglitazone prevented these abnormalities through the PPAR-γ/PGC-1α pathway.

## Introduction

Atrial fibrillation (AF) is the most prevalent sustained cardiac arrhythmia observed in clinical practice, and its prevalence increases with increasing age and is associated with increased morbidity and mortality (Chugh et al., 2014). To date, medical treatment for preventing AF remains unsatisfactory (Chugh et al., 2014). Of the different risk factors of AF, diabetes mellitus is one of the more prevalent ones (Goudis et al., 2015). Although the exact pathophysiological mechanisms linking diabetes to AF development have not been completely elucidated, numerous lines of evidence suggest that oxidative stress and inflammation may act as central mediators (Karam et al., 2017).

Oxidative stress is mainly related to excessive production of reactive oxygen species (ROS). The imbalance between ROS scavenging and generation promotes oxidative stress and inflammation. ROS can react with multiple cellular components (i.e., lipids, proteins, and DNA) and is associated with DNA damage, apoptosis, and cardiac hypertrophy and fibrosis (Finkel and Holbrook, 2000). The mitochondria are the most important cellular source of ROS in most mammals, and hyperglycemic conditions increase mitochondrial ROS production (Bhatti et al., 2017). Excessive ROS production damages mitochondrial proteins and DNA, leading to the disruption of ATP production and other essential functions in the mitochondria (Bhatti et al., 2017).

Pioglitazone, an agonist of peroxisome proliferator-activated receptor γ (PPAR-γ) that also causes partial activation of PPAR-α, improved cardiovascular outcome in patients who did not have diabetes but who had insulin resistance and in patients with type 2 diabetes mellitus (Dormandy et al., 2005; Kernan et al., 2016). Our previous studies suggested that oxidative stress and inflammation play an important role in diabetic atrial remodeling, and that pioglitazone attenuates diabetes-induced atrial structural and electrical remodeling through antioxidant and anti-inflammatory actions (Liu et al., 2017). In the present study, we evaluated the role of mitochondrial oxidative stress in atrial remodeling in diabetes and the potential effects of pioglitazone.

## Materials and Methods

### Animal Studies

The animal protocols were approved by the Experimental Animal Administration Committee of Tianjin Medical University and the Tianjin Municipal Commission for Experimental Animal Control. All animal experiments were performed in accordance with U.S. National Institutes of Health guidelines. The animal work took place at the Animal Center of the Tianjin Institute of Cardiology and the Tianjin University of Sport. All experimental animals were killed by quickly isolating their hearts under full anesthesia with 3% sodium pentobarbital.

For this study, Japanese white rabbits of either sex weighing between 1.9 and 2.5 kg were randomly divided into three groups: normal control (C group), diabetic (Diabetic group), and pioglitazone-treated diabetic (Pio group). Our team has previously conducted experimental studies using a diabetes model in rabbits (Fu et al., 2016; Qiu et al., 2017; Zhou et al., 2021). Briefly, diabetes was induced by injecting 120 mg/kg of 5% alloxan monohydrate (Sigma, St. Louis, MO, United States) freshly dissolved in sterile normal saline *via* the marginal ear vein (Zhang et al., 2018). Fasting blood glucose levels were determined 48 h later using a glucometer (Abbott, Bedford, MA, United States), and the presence of diabetes mellitus was confirmed by blood glucose levels ≥14 mmol/L (once) or ≥11 mmol/L (twice). Rabbits in the Pio group received 4 mg/kg/day of pioglitazone (Tianjin Takeda Pharmaceuticals Co., Ltd., Tianjin, China) orally (Liu et al., 2017); all animals were kept in cages and received water and standard laboratory pellet diet during the 8-week study period.

### Echocardiographic Measurements

After 8 weeks, transthoracic echocardiography was performed by blinded operators. The rabbits were anesthetized with 3% sodium pentobarbital (30 mg/kg) and their chest area was shaved, and the rabbits were placed on the table in the left lateral decubitus position. Images were acquired with a GE Vingmed machine (Vivid 7/Vingmed General Electric, Milwaukee, WI, United States) with a 7.5-MHz standard pediatric probe. Two-dimensional and M-mode images of the left ventricular (LV) long axis view were used for measuring the chamber size and wall diameter. The LV ejection fraction (LVEF) was calculated according to our previous study (Fu et al., 2015).

### Surface Electrocardiographic and Hemodynamic Studies

After 8 weeks, surface electrocardiogram (ECG) tracing was obtained in the rabbits under 3% sodium pentobarbital anesthesia using the BL-420F biological function detection system (Chengdu Taimeng Science and Technology Co., Ltd., Chengdu, China). Subsequently, the right carotid artery was isolated surgically, and a cannula was inserted to measure the aortic systolic and diastolic blood pressure (SBP and DBP, respectively); then, the cannula was advanced into the left ventricle through the aortic valve to detect the LV end-diastolic pressure (LVEDP) and the maximum rate of LV pressure rise and fall (±dP/dt_max_).

After the recording, blood samples were obtained from the postcava for measuring the plasma parameters. Then, the animals were euthanized, and the left atrial (LA) tissues were immediately collected, frozen in liquid nitrogen, and stored at −80°C. Furthermore, small pieces of LA tissue were immersed in 4% paraformaldehyde for histological studies.

### Electrophysiological Study

The rabbits were anesthetized with 3% sodium pentobarbital. The hearts were quickly removed and placed in cold perfusion fluid (4°C), and then, the aorta was cannulated and connected to a Langendorff perfusion system filled with 37°C Tyrode’s solution equilibrated with 5% CO_2_ and 95% O_2_. The perfusion pressure was maintained at 80–95 mmHg. Four silver bipolar electrodes were placed on the high right atrium (HRA), high left atrium (HLA), low left atrium (LLA), and right ventricular apex (RV), and then connected to a custom-made computer software program (Electrophysiological Recording System, TOP-2001, HTONG Company, Shanghai, China). Eight basic stimuli (S1) were followed by a premature extra stimulus (S2), and the S1S1 cycle had three basic cycle lengths (BCLs; 150, 200, and 250 ms). The S1S2 interval was decreased in 2-ms steps until the atrial effective refractory period (AERP) was reached, which was defined as the longest S1S2 interval that failed to induce an action potential. AERP dispersion (AERPD) was defined as the difference between the longest and shortest AERP at three different sites (HRA, HLA, and LLA). Interatrial conduction time (IACT) was defined as the duration from the HRA pacing stimulus to the beginning of the HLA stimulus. The Wenckebach cycle length of atrial–ventricular conduction (AVWCL) was measured by right atrial incremental pacing. AF inducibility was tested by the application of burst pacing (cycle length of 50 ms) for 1 s, five times with 30-s intervals at the HRA, HLA, and LLA. AF was defined as rapid and irregular atrial excitations lasting for ≥1 s.

### Measurement of Serum Parameters

Blood samples were drawn *via* the postcava before the rabbits were euthanized. The samples were centrifuged at 3,000 rpm for 10 min within 30 min of collection to separate the serum. The serum samples were stored at -80°C in a refrigerator and thawed at room temperature before use. Rabbit enzyme–linked immunosorbent assay (ELISA) kits were used to detect the serum levels of insulin (Wuhan Huamei Biological Engineering Co., Ltd., Hubei, China), PPAR-α and PPAR-γ (Shanghai Huding Biological Technology Co., Shanghai, China), and several inflammation and oxidative stress markers; these included high-sensitivity C-reactive protein (hs-CRP, Wuhan Huamei Biological Engineering Co., Ltd.), lipid peroxidation of malondialdehyde (MDA, Nanjing Jiancheng Bioengineering Institute, Jiangsu, China), 8-hydroxy-2 deoxyguanosine (8-OHdG, Shanghai Huding Biological Technology Co.), and the activity of total superoxide dismutase (SOD, Nanjing Jiancheng Bioengineering Institute).

### Histological and Immunohistochemical Determination

Standardized H&E and Masson’s trichrome stains were performed as reported previously (Zhang X. et al., 2017). After the hemodynamic study, isolated LA tissue was fixed in 4% paraformaldehyde for 3 days and then embedded in paraffin. The LA myocardium was cut into 4-µm cross-sections, deparaffinized, rehydrated, and stained with H&E and Masson’s trichrome stains to determine the cardiomyocyte diameter and interstitial fibrosis, respectively. Photographs were captured with an Olympus BX-53 microscope (Olympus, Tokyo, Japan) and analyzed using Image-Pro Plus 6.0 Scion image software (Scion Co., Frederick, MD, United States). Five randomly selected sections from each myocardial preparation were utilized for quantification. In each rabbit, the atrial myocyte diameter was measured as the average of 40 cardiomyocytes, with the nucleus in the plane of the section. The fibrous tissue content was quantified as the percentage of blue-stained areas relative to the total tissue area, excluding the perivascular fibrotic regions.

### Electron Microscopy

After a 2-h fixation in 2.5% glutaraldehyde, LA tissues were further fixed in 1% osmium tetroxide, dehydrated in ethanol, and embedded in epoxy resin. Ultrathin sections were cut from each sample, counterstained with uranyl acetate and lead citrate, and observed using an H-7650 transmission electron microscope (Hitachi, Tokyo, Japan).

### Mitochondrial Isolation

After the rabbits had been sacrificed under 3% sodium pentobarbital anesthesia, the atrial tissue was rapidly dissected and minced in ice-cold isolation medium (pH 7.4) containing 220 mmol/L mannitol, 70 mmol/L sucrose, 5 mmol/L HEPES, 1 mmol/L PMSF, and 0.2% (w/v) bovine serum albumin (BSA). The tissue was homogenized using a manual glass homogenizer under six passes (0–4°C). Subsequently, the homogenate was centrifuged at 1,000 × g for 10 min, and then the supernatant was centrifuged at 10,000 × g for 10 min to obtain the mitochondrial pellet. Then, the mitochondrial pellet was suspended in 0.5 ml conversational medium (pH 7.4) containing 220 mmol/L mannitol, 70 mmol/L sucrose, and 5 mmol/L HEPES.

Mitochondrial protein concentration was quantified using the Bradford colorimetric method. The mitochondrial isolation procedures were completed within 1 h after the rabbits had been sacrificed.

### Mitochondrial Respiration Assay

Mitochondrial respiratory function was assessed in isolated mitochondria polarographically at 25°C, by measuring O_2_ consumption using an Oroboros Oxygraph (Oroboros Instruments, Innsbruck, Austria). In a 2-ml, closed, thermostatic, magnetically stirred glass chamber, respiration medium (225 mmol/L mannitol, 70 mmol/L sucrose, 1 mmol/L EDTANa_2_, 20 mmol/L KH_2_PO_4_, and 1 mg/ml BSA, pH 7.4) was saturated with ambient oxygen to reach a concentration of 258 μmol/L. After standardized instrument and chemical calibrations, 300 μg of isolated mitochondria were added to the reaction system. State 2 respiration was assessed with the addition of a 15-μl mixture of 0.8 mol/L malic acid and 1 mol/L glutamic acid, and then, state 3 respiration was assessed by the addition of ADP (0.5 mM). After all of the ADP had been phosphorylated to ATP, the respiratory rate turned to state 4. The respiratory control ratio (RCR) was calculated as the respiratory rate in state 3 divided by that in state 4.

### Mitochondrial Membrane Potential and ROS Production Measurements

The mitochondrial membrane potential (MMP, ∆Ψm) was examined *via* JC-1 staining (Beyotime, Jiangsu, China). Mitochondria (300 μg) in 2 ml respiration medium were incubated with JC-1 working solution at 37°C in the dark for 10 min, and then, mitochondrial respiratory function was initiated by a 15-μl mixture of 0.8 mol/L malic acid and 1 mol/L glutamic acid. The alteration of the fluorescence emission was detected using a Cary Eclipse fluorescence spectrophotometer (Varian, Palo Alto, CA, United States).

The mitochondrial ROS generation was measured fluorometrically using 2′, 7′-dichlorodihydrofluorescein diacetate (DCFH-DA, Sigma). Briefly, 300 μg of isolated mitochondria were incubated with fresh incubation medium containing 5 μM DCFH-DA at 37°C in the dark for 15 min. Fluorescence was determined for 2 min at 499 nm for excitation and 521 nm for emission using a Cary Eclipse fluorescence spectrophotometer (Varian).

### HL-1 Cell Culture and Small Interfering RNA Transfection

HL-1 atrial cardiomyocytes derived from adult mouse atria were cultured in Claycomb medium (Sigma) according to the developer’s instructions (Claycomb et al., 1998). Once they had reached 70–80% confluence, passage 3–4 cells were transfected with PGC-1α siRNA or control siRNA (nonspecific scrambled siRNA) with Lipofectamine 3000 (Invitrogen) for 24 h as per the manufacturer’s instructions. After siRNA transfection, the cells were pretreated with vehicle or pioglitazone (10 μM, Sigma) (Gu et al., 2013) for 1 h and then stimulated with 300 μM H_2_O_2_ for 24 h. The cells were randomly divided into the following groups: control siRNA, control siRNA + H_2_O_2_, control siRNA + H_2_O_2_ + Pio, PGC-1α siRNA, PGC-1α siRNA + H_2_O_2_, and PGC-1α siRNA + H_2_O_2_ + Pio.

### MMP Measurement of HL-1 Cells

The HL-1 cells were incubated with JC-1 working solution at 37°C for 20 min and washed twice with phosphate-buffered saline (PBS). MMP was determined *via* the relative amounts of dual emissions from mitochondrial JC-1 aggregates or monomers using an Olympus FV1000 confocal microscope under 543 and 488 nm laser excitation. A decrease in the densitometry ratio of aggregate JC-1 red/monomeric JC-1 green indicated a decrease in the MMP. Data are expressed as a normalized ratio of the fold change to the control group.

### Western Blotting

Proteins from LA tissues or HL-1 cells were pulverized in liquid nitrogen and extracted using radioimmunoprecipitation lysis buffer. The protein content was assayed using a bicinchoninic acid protein assay reagent kit (Thermo Fisher Scientific, Waltham, MA, United States). Protein samples (60 μg) were separated by sodium dodecyl sulfate–polyacrylamide gel electrophoresis (SDS-PAGE), transferred to PVDF membranes (Millipore, Bedford, MA, United States), and blocked with 5% milk or BSA. Subsequently, the membranes were incubated overnight at 4°C with primary antibodies against nuclear factor kappa B (NF-κB; Abcam, Cambridge, MA, United States; ab90523), transforming growth factor β1 (TGF-β1; Abcam, ab190503), PGC-1α (Abcam, ab54481), nuclear respiratory factor 1 (NRF1; Santa Cruz, Heidelberg, Germany; sc-101102), mitochondrial transcription factor A (TFAM; Novus Biologicals, Littleton, CO, United States; NBP2-19437), optic atrophy 1 (Opa1; Abcam, ab157457), mitofusin 1 (Mfn1; Abcam, ab104274), Mfn2 (Abcam, ab56889), and dynamin-related protein 1 (Drp1; Abcam, ab140494). After rinsing in Tris-buffered saline-Tween 20 (TBST) buffer, the membranes were reacted with horseradish peroxidase–conjugated secondary antibody. β-Actin (TransGen Biotech Co., Ltd., Beijing, China) was used as a loading control. Band intensity was detected using a Tanon 5,200 Multi Chemiluminescent Imaging System (Tanon Science & Technology Co., Ltd., Shanghai, China) and quantified by densitometry.

### Statistical Analysis

Data are expressed as the mean ± SEM. Comparison groups were performed using one-way analysis of variance (ANOVA) with the Bonferroni correction. The inducibility of AF was analyzed by using Fisher’s exact test. *p < 0.05* was considered statistically significant. Statistical analysis was performed using SPSS 22.0 statistical software (SPSS, Chicago, IL, United States).

## Results

The characteristics of the included animals are shown in Table 1. After 8 weeks, the diabetic group and the Pio group demonstrated higher blood glucose and lower blood insulin levels than the control.

**TABLE 1:** Metabolic, hemodynamic, and echocardiographic parameters of the control group (C group), diabetic group (Diabetic group), and pioglitazone-treated diabetic group (Pio group).

	C group (*n* = 8)	Diabetic group (*n* = 8)	Pio group (*n* = 8)	*p* value
Glucose (mmol/L)	5.25 ± 0.33	15.99 ± 2.46^**^	16.84 ± 2.93^**^	0.002
Insulin (mIU/L)	16.7 2 ± 1.12	6.53 ± 0.84^**^	6.79 ± 0.77^**^	<0.001
Weight (kg)	3.09 ± 0.10	2.57 ± 0.17	2.86 ± 0.20	0.096
HR (bpm)	295.63 ± 8.12	294.75 ± 8.37	292.63 ± 13.09	0.977
SBP (mmHg)	107.70 ± 4.30	117.89 ± 3.74	112.25 ± 6.70	0.382
DBP (mmHg)	96.61 ± 3.31	101.72 ± 3.49	99.38 ± 6.16	0.729
MBP (mmHg)	100.32 ± 3.63	108.19 ± 3.68	103.68 ± 6.29	0.504
LVEDP (mmHg)	5.80 ± 3.31	7.51 ± 2.40	5.67 ± 2.09	0.861
+ dp/dt_max_ (mmHg/m)	2,580.7 ± 438.7	3,554.8 ± 734.9	2,856.6 ± 191.4	0.391
- dp/dt_max_ (mmHg/m)	2,282.1 ± 328.9	3,075.3 ± 552.8	2,555.7 ± 167.8	0.351
LAD (mm)	7.05 ± 0.28	8.73 ± 0.33^**^	7.47 ± 0.28^#^	0.003
IVS (mm)	1.71 ± 0.08	2.30 ± 0.12^*^	1.95 ± 0.20^*^	0.025
LVPW (mm)	1.68 ± 0.06	2.35 ± 0.11^**^	2.29 ± 0.08^**^	<0.001
LVEDD (mm)	12.21 ± 0.29	13.41 ± 0.51	11.73 ± 0.54	0.047
LVESD (mm)	7.12 ± 0.25	7.90 ± 0.34	7.34 ± 0.34	0.220
LVEF (%)	63.07 ± 1.81	60.55 ± 2.16	57.50 ± 1.67	0.139

Values are mean ± SEM; HR, heart rate; SBP, systolic blood pressure; DBP, diastolic blood pressure; MBP, mean blood pressure; LVEDP, left ventricular end diastolic presssure; + dp/dt_max_, maximal increasing rate of left intraventricular pressure; - dp/dt_max_, maximal decreasing rate of left intraventricular pressure; LAD, left atrial diameter; IVS, interventricular septa; LVPW, left ventricular posterior wall; LVEDD, left ventricular end-diastolic dimension; LVESD, left ventricular end-systolic dimension; LVEF, left ventricular ejection fraction. *Compared with the C group, p < 0.05; **compared with the C group, p < 0.01; ^#^compared with the Diabetic group, p < 0.05.

### Pioglitazone Attenuated Diabetes-Induced Atrial Structural Remodeling

**FIGURE 1:**
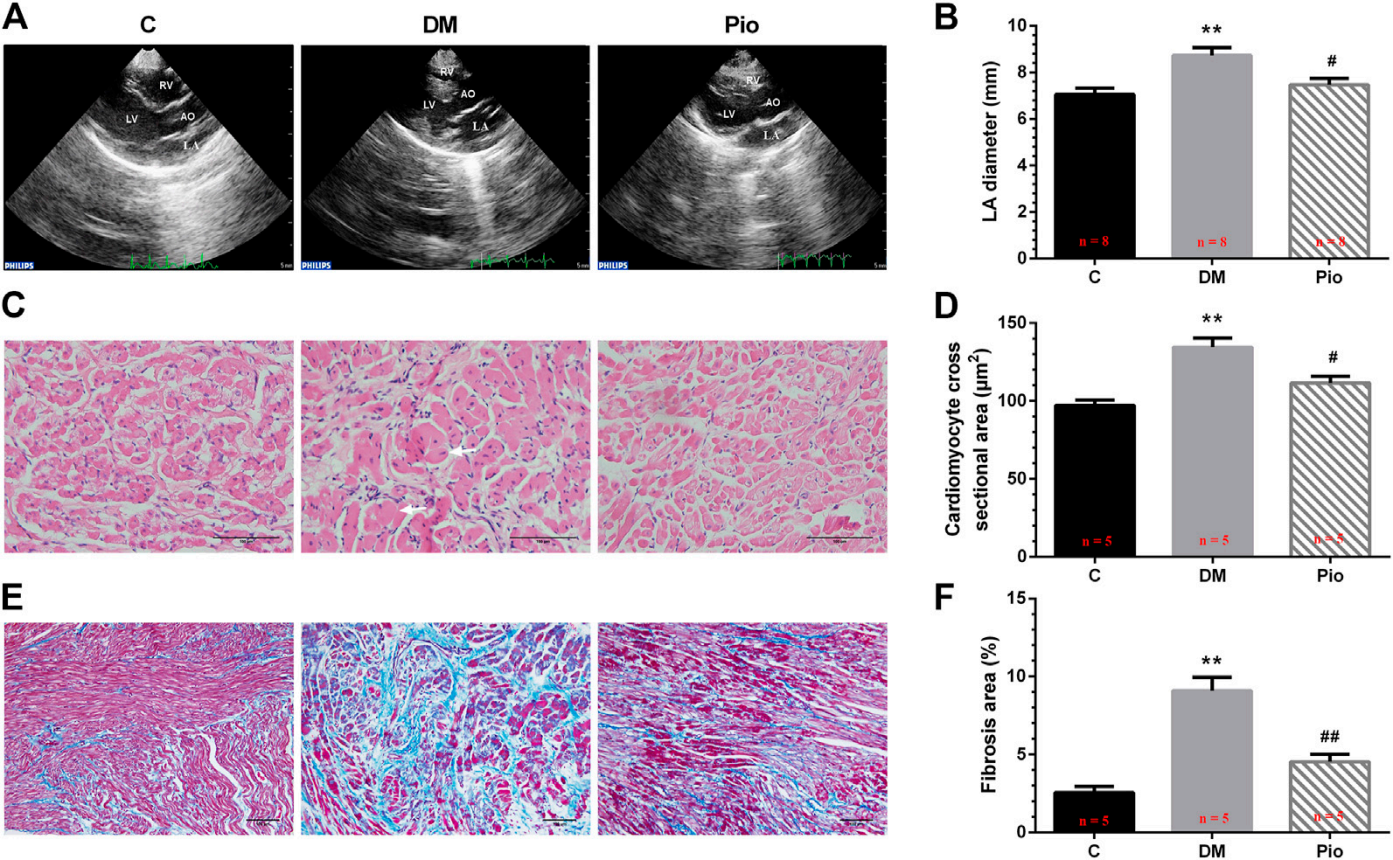
Pioglitazone attenuated diabetes-induced atrial structural remodeling. Representative echocardiographic images **(A)**, left atrial diameter **(B)**, H&E staining (400× magnification) **(C)**, atrial cardiomyocyte diameter **(D)**, Masson’s trichrome staining (200× magnification) **(E)**, and interstitial fibrosis **(F)** from the control, diabetes mellitus (DM), or pioglitazone-treated DM (Pio) groups. LA, left atrium; LV, left ventricle; RV, right ventricle; and AO, aorta. Arrows indicate hypertrophic atrial myocytes in diabetic rabbits. Values are presented as mean ± SEM. **Compared with the C group, *p* < 0.01; ^#^compared with the Diabetic group, *p* < 0.05; ^##^compared with the Diabetic group, *p* < 0.01. *n* = 5–8 per group.

Representative echocardiographic images of the control, diabetic, and pioglitazone-treated diabetic rabbits are shown in Figure 1A. The LA diameter (LAD) (Figure 1B), interventricular septa (IVS), and LV posterior wall (LVPW) were significantly higher in the Diabetic group (Table 1) than they were in the control group. No significant differences in SBP or DBP, LVEDP, and hemodynamic parameters were observed (Table 1). Pioglitazone prevented the diabetes-induced increase in LAD while partially ameliorating the changes in the IVS and LVPW. Figure 1C shows H&E-stained (400× magnification) images; the Diabetic group had a higher cardiomyocyte cross-sectional area (Figure 1D). Figure 1E show Masson’s trichrome staining images (200× magnification); the Diabetic group had a greater degree of LA interstitial fibrosis (Figure 1F). Pioglitazone treatment reversed these histological abnormalities.

### Pioglitazone Inhibited Diabetes-Induced Electrical Remodeling and Prevented the Development of AF

**FIGURE 2:**
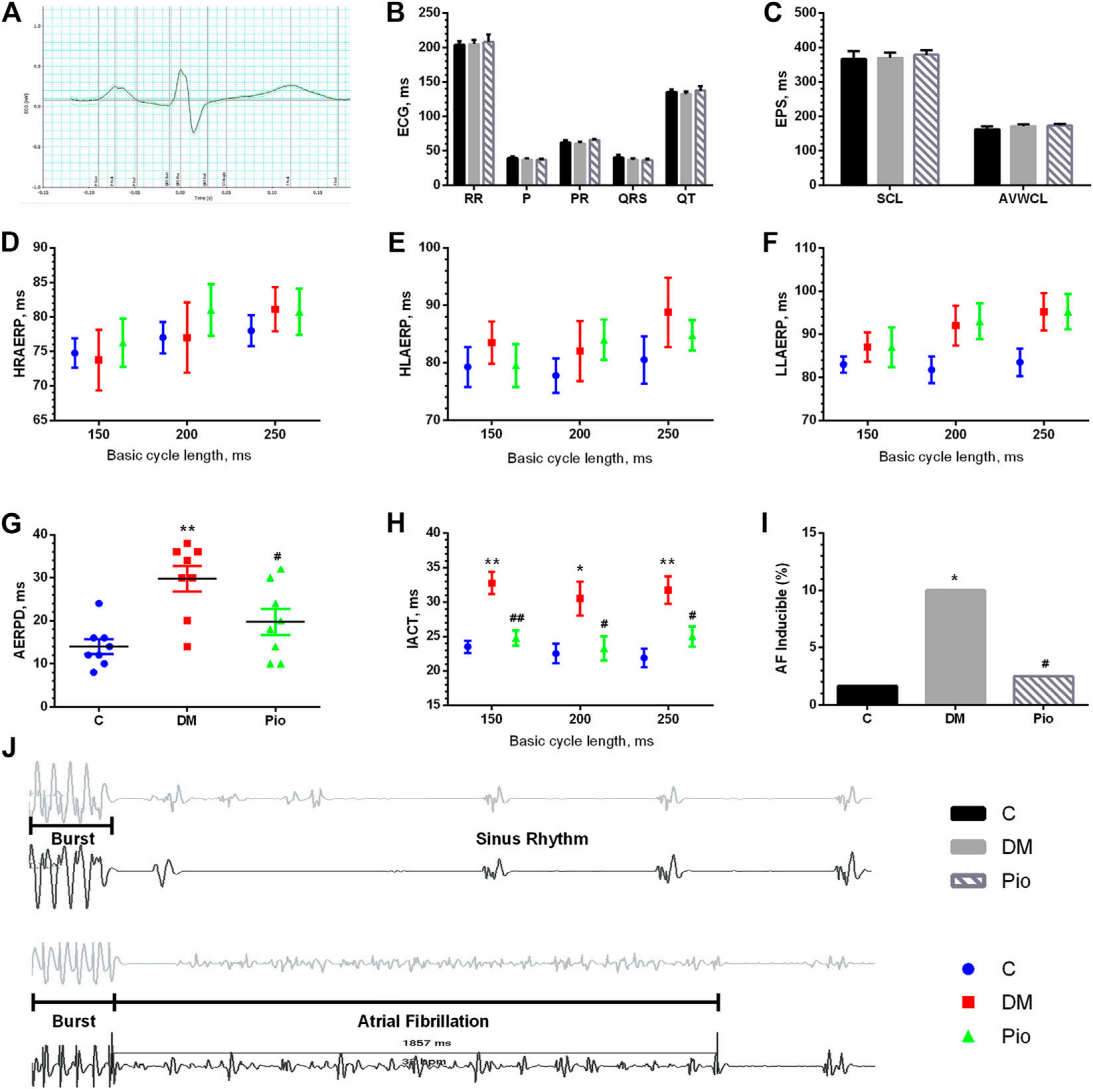
Pioglitazone inhibited diabetes-induced electrical remodeling and prevented the development of AF. An example electrocardiogram trace **(A)** and quantification of ECG parameters (RR interval, P-wave duration, PR interval, QRS duration, and QT intervals) **(B)**. **(C)** Parameters from EPS [sinus cycle length (SCL) and AV Wenckebach cycle length (AVWCL)]. **(D–F)** Quantifications of high right atrium effective refractory period (HRAERP), high left atrium effective refractory period (HLAERP), and low left atrium effective refractory period (LLAERP) at basic cycle lengths (BCLs) of 150, 200, and 250 ms. **(G)** Quantification of atrial effective refractory period dispersion (AERPD). **(H)** Interatrial conduction time at BCLs of 150, 200, and 250 ms. **(I)** Quantification of the inducibility of AF. **(J)** Representative AF episode and control sinus rhythm data induced by burst pacing. Values are presented as mean ± SEM. *Compared with the C group, *p* < 0.05; **compared with the C group, *p* < 0.01; #compared with the Diabetic group, *p* < 0.05; ^##^compared with the Diabetic group, *p* < 0.01. *n* = 8 per group.

We performed surface ECG and EPS to determine whether pioglitazone inhibited diabetes-induced atrial electrical remodeling. Figure 2A depicts a sample electrocardiogram. The RR interval, P wave duration, PR interval, QRS duration, and QT intervals were not significantly different among the three groups (Figure 2B). The EPS data also illustrated no difference in the SCL and AVWCL (Figure 2C). The AERP values from the HRA, HLA, and LLA were not significantly different among the three groups at the BCLs of 150, 200, and 250 ms, respectively (Figures 2D–F). By contrast, the AERPD (Figure 2G) and IACT (Figure 2H) were significantly higher in the Diabetic group—changes that were attenuated by pioglitazone treatment. These electrophysiological changes were associated with a higher incidence of inducible AF by burst pacing in the Diabetic group than in the control group (Figure 2I), which pioglitazone treatment markedly reduced (Figure 2I). Figure 2J illustrates the representative AF episode and control sinus rhythm data induced by the burst pacing.

### Serum PPAR-γ, PPAR-α, and Oxidative Stress and Inflammatory Parameters

**FIGURE 3:**
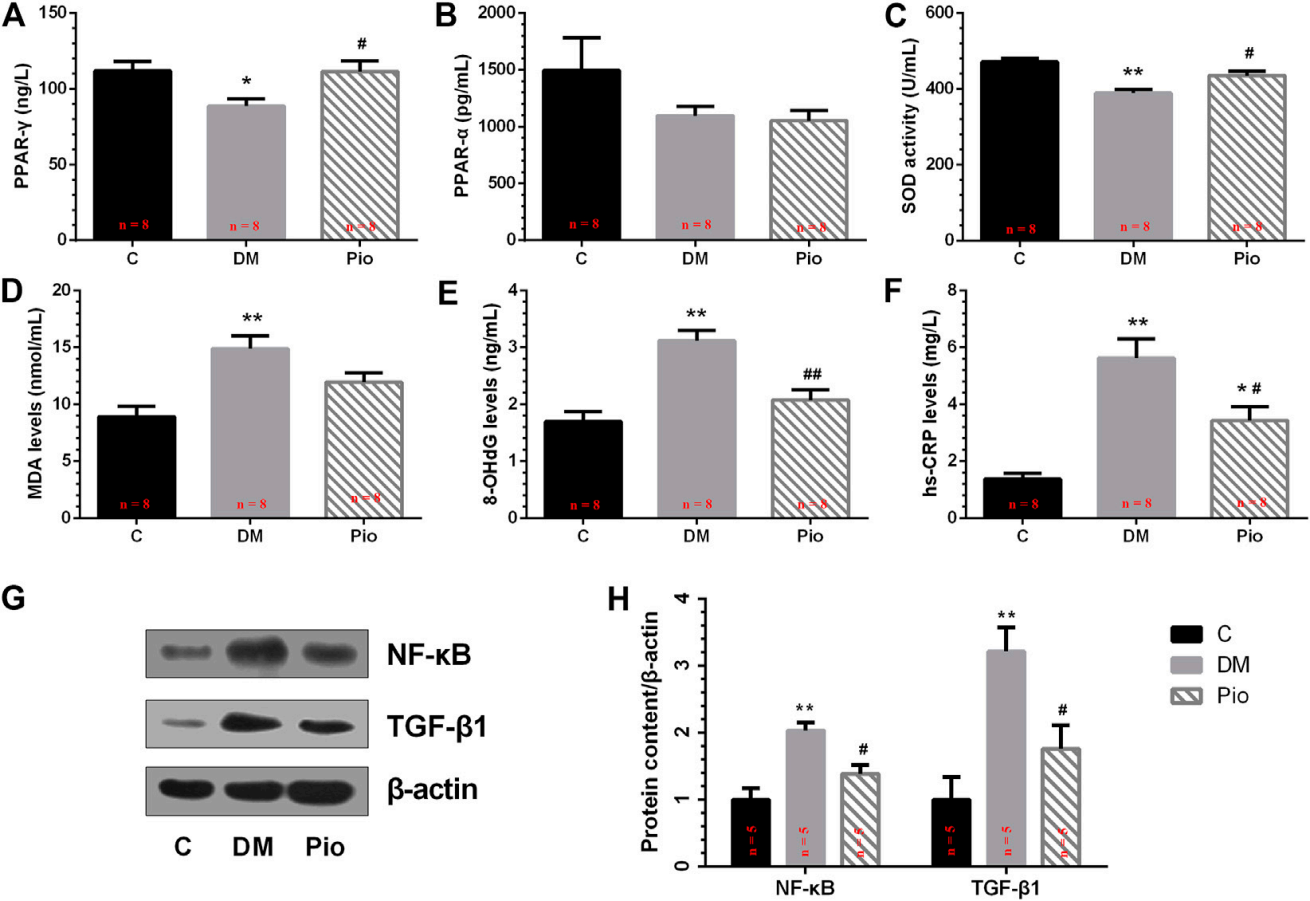
Effects of pioglitazone on PPAR-γ, PPAR-α, and oxidative stress and inflammatory parameters in serum **(A–F)** and left atrium **(G,H)**. Values are presented as mean ± SEM. *Compared with the C group, *p* < 0.05; **compared with the C group, *p* < 0.01; ^#^compared with the Diabetic group, *p* < 0.05; ^##^compared with the Diabetic group, *p* < 0.01. *n* = 5–8 per group.

We quantified the serum PPAR-γ levels, which were significantly lower in the Diabetic group than in the controls, whereas the pioglitazone treatment returned the levels to normal (Figure 3A). By contrast, there was no significant difference in PPAR-α among the three groups (Figure 3B). Next, the oxidative stress and inflammation markers in the serum were quantified. The Diabetic group had significantly lower SOD activity (Figure 3C) and higher MDA (Figure 3D), 8-OHdG (Figure 3E), and hs-CRP (Figure 3F) levels than the controls. Pioglitazone prevented changes in these parameters, apart from MDA.

### Left Atrial NF-κB and TGF-β1 Protein Expression

To examine the effects of pioglitazone on oxidative stress and inflammatory changes, we determined the NF-κB and TGF-β1 protein expression levels in the left atrium (Figure 3G). Diabetes increased NF-κB and TGF-β1 expression levels, and pioglitazone treatment prevented these changes (Figure 3H).

### Pioglitazone Inhibited Diabetes-Induced Mitochondrial Oxidative Stress and Dysfunction

**FIGURE 4:**
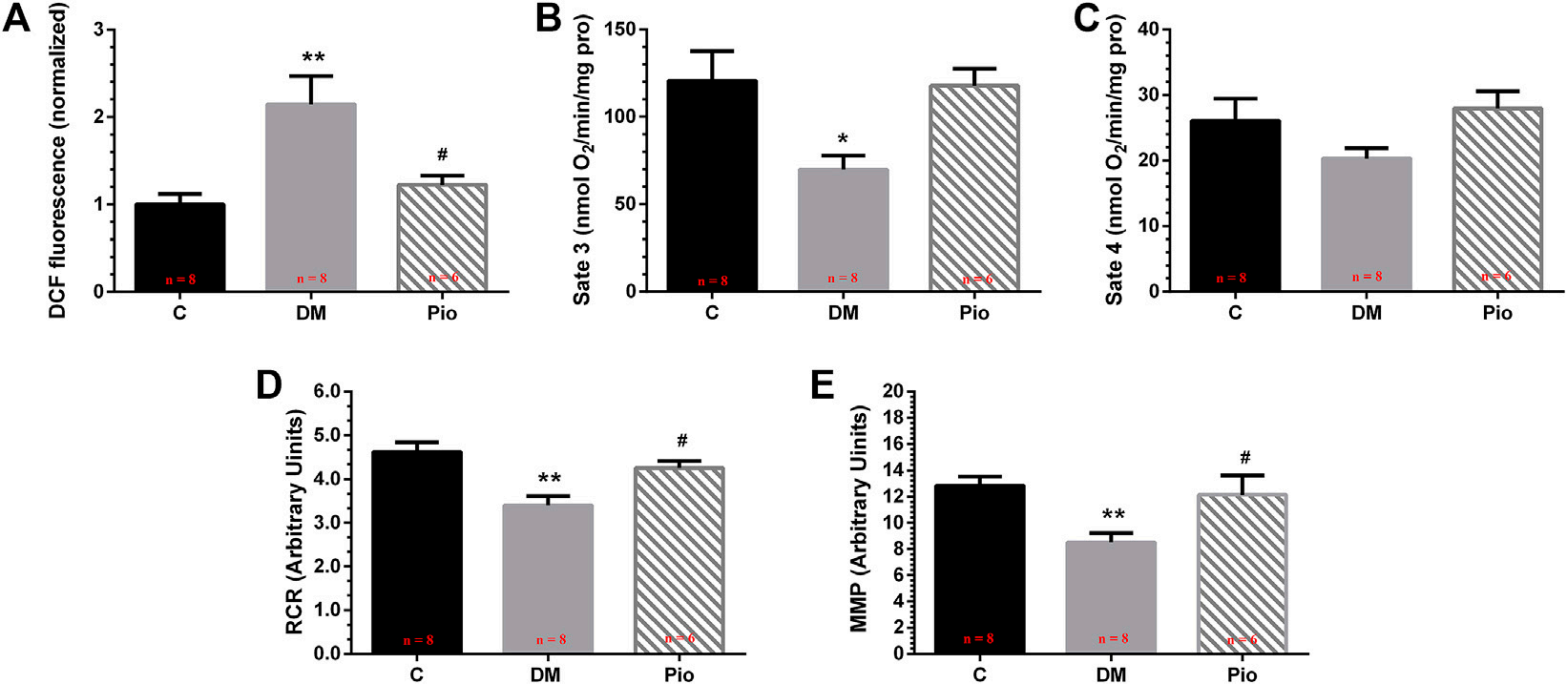
Pioglitazone inhibited diabetes-induced mitochondrial oxidative stress and dysfunction. Quantification of mitochondrial reactive oxygen species (ROS) levels **(A)**, state 3 respiration rate **(B)**, state 4 respiration rate **(C)**, respiratory control ratio (RCR) **(D),** and mitochondrial membrane potential (MMP) **(E)**. Values are presented as mean ± SEM. *Compared with the C group, *p* < 0.05; **compared with the C group, *p* < 0.01; #compared with the Diabetic group, *p* < 0.05. *n* = 6–8 per group.

We investigated the mitochondrial function of LA cardiomyocytes. The dichlorofluorescein (DCF) assay detected higher mitochondrial ROS generation (Figure 4A) and lower state 3 and 4 respiratory rates (Figures 4B,C), which corresponded to a lower RCR (Figure 4D) and lower MMP (Figure 4E) in the Diabetic group. Pioglitazone treatment prevented these changes.

### Pioglitazone Improved Diabetes-Induced Mitochondrial Network Damage and the Dynamic Balance Between Fusion and Fission

**FIGURE 5:**
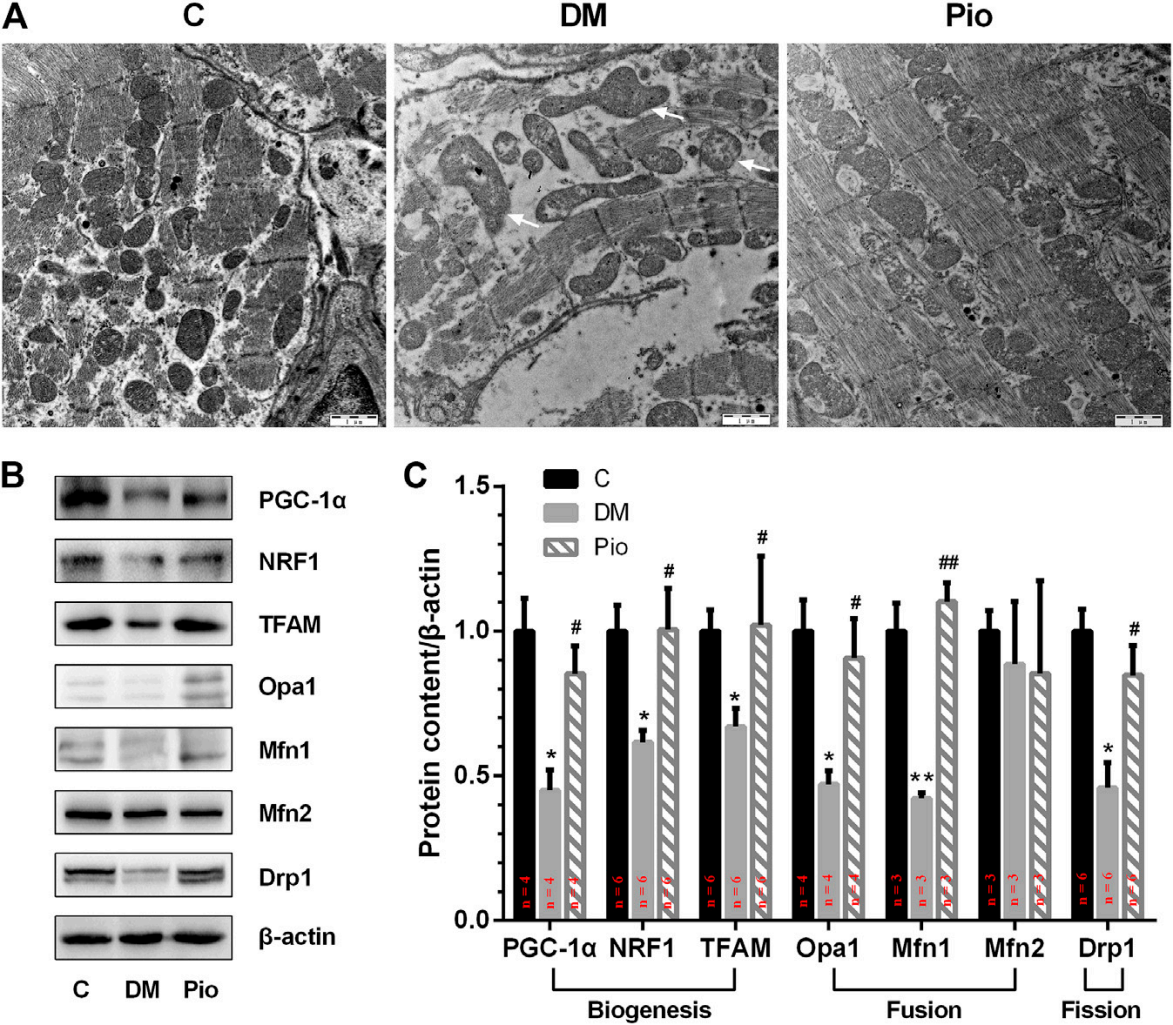
Pioglitazone improved diabetes-induced mitochondrial network damage and the dynamic balance between mitochondrial fusion and fission. **(A)** Comparison of left atrial ultrastructure among the three groups. Diabetic rabbits present severely disintegrated myofilaments and swelling of mitochondria, accompanied by fractured cristae (arrows), which could be ameliorated by pioglitazone treatment. **(B)** Expression of proteins involved in mitochondrial biogenesis, fusion, and fission assessed by Western blotting analysis. **(C)** Quantifications of protein expression of **(B)**. Values are presented as mean ± SEM. *Compared with the C group, *p* < 0.05; **compared with the C group, *p* < 0.01; #compared with the Diabetic group, *p* < 0.05; ##compared with the Diabetic group, *p* < 0.01. *n* = 3–6 per group.

Electron microscopic imaging of the LA tissue sections demonstrated regular sarcomere organization and uniformly sized mitochondria between sarcomeres in the control group (Figure 5A). By contrast, the diabetic state led to abnormal mitochondrial morphology, including severely disintegrated myofilaments and swelling, accompanied by fractured cristae. Pioglitazone treatment partly improved the diabetes-induced mitochondrial network damage.

Next, we tested the hypothesis that the diabetes-induced mitochondrial injury is associated with an imbalance in mitochondrial dynamics, which could be resolved by pioglitazone. PGC-1α, which plays a critical role in controlling mitochondrial biogenesis and function, was significantly reduced in the Diabetic group compared with the control (Figures 5B,C). The expression of other mitochondrial biogenesis-related proteins, that is, NRF1 and TFAM, was also significantly decreased. Moreover, the levels of the fusion-related proteins (Opa1 and Mfn1) and the fission-related protein (Drp1) were also significantly lower in the Diabetic group. However, pioglitazone treatment reversed these changes. No significant differences in the expression of the fusion-related protein Mfn2 were detected among the three groups.

### Knockdown of PGC-1α Blunted the Effects of Pioglitazone on Mn-SOD Protein Expression and MMP Collapse in H_2_O_2_-Treated HL-1 Cells

**FIGURE 6:**
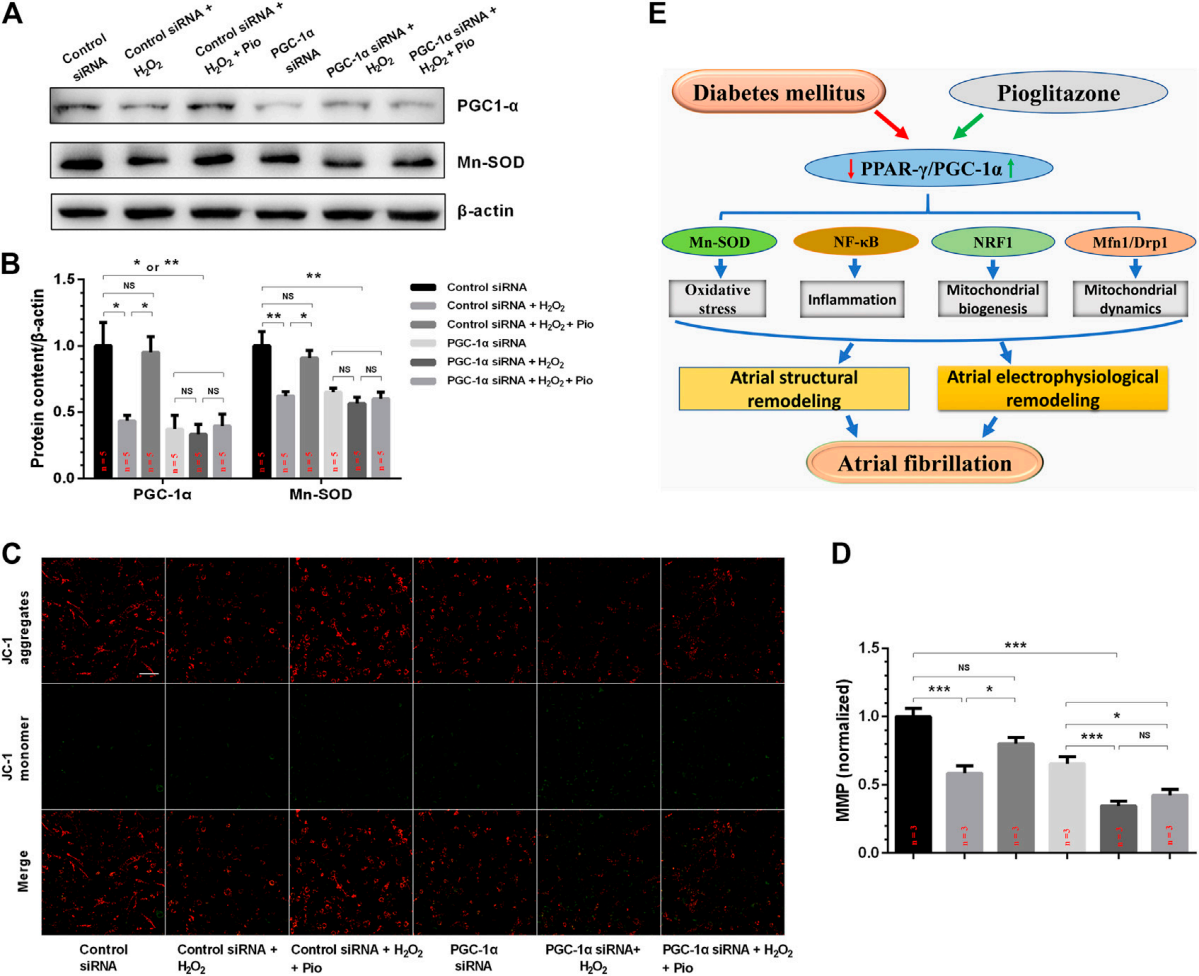
PGC-1α siRNA blunted the effect of pioglitazone (Pio) on Mn-SOD protein expression and mitochondrial membrane potential (MMP) collapse in H_2_O_2_-treated HL-1 cells. **(A)** Representative Western blotting results. **(B)** Quantification of protein expression. Values are presented as mean ± SEM. * *p* < 0.05; ** *p* < 0.01; NS, not significant. *n* = 5 independent experiments. **(C)** Representative confocal microscopy images of HL-1 cells that were stained with JC-1 dye. **(D)** Quantification of **(C)**. *n* = 3 independent experiments (scale bar = 100 μm). **(E)** Schematic figure illustrating that pioglitazone prevents diabetes-induced mitochondrial oxidative stress and atrial remodeling through the PPAR-γ/PGC-1α pathway.

To examine whether pioglitazone inhibits mitochondrial oxidative stress and improves mitochondrial function through the PPAR-γ/PGC-1α signaling pathway, we transfected HL-1 cells with PGC-1α siRNA and analyzed the changes in mitochondrial oxidative stress and function. Figures 6A,B show that, in contrast to the control siRNA cells, H_2_O_2_-treated cells had significantly lower PGC-1α and Mn-SOD protein levels, and pioglitazone treatment prevented these changes. However, no difference was observed between the groups transfected with PGC-1α siRNA. Similarly, knocking down PGC-1α also blunted the effects of pioglitazone in preserving MMP in HL-1 cells under oxidative stress (Figures 6C,D).

## Discussion

The main findings of the present study are 1) pioglitazone protected against diabetes-induced atrial structural and electrophysiological remodeling; 2) these proactive effects were associated with decreased TGF-β1 protein expression, higher SOD activity, reduced atrial mitochondrial ROS production, and suppressed oxidative and inflammatory marker expression in serum (8-OHdG and hs-CRP) and LA (NF-κB) in diabetic rabbits; 3) pioglitazone ameliorated diabetes-induced atrial mitochondrial swelling, prevented mitochondrial respiratory dysfunction, and preserved MMP and alterations in biogenesis (PGC-1α, NRF1, and TFAM)-, fusion (Opa1 and Mfn1)-, and fission (Drp1)-related protein expression; and 4) pioglitazone mediates these beneficial effects through the PPAR-γ/PGC-1α signaling pathway (Figure 6E).

Diabetes is a well-recognized risk factor for the development of AF (Xiong et al., 2018), adverse cardiovascular events (Lee et al., 2021a; Lee et al., 2021b), complications involving different organs (Lee et al., 2020), and mortality. Of the different classes of antidiabetic drugs, thiazolidinediones act by activating PPAR-γ (Lebovitz, 2019; Veettil et al., 2020). Pioglitazone is a thiazolidinedione with favorable effects on insulin sensitivity, blood glucose, lipid metabolism, inflammation, and oxidative stress (DeFronzo et al., 2019). Concerns have been raised regarding potential adverse effects resulting from thiazolidinedione use, such as heart failure and bladder cancer, leading to the discontinuation of these drugs in some European countries. However, the recent results of the IRIS (Insulin Resistance Intervention after Stroke) trial did not demonstrate higher incidences of heart failure and bladder cancer with pioglitazone treatment (Kernan et al., 2016). Moreover, the IRIS trial (Kernan et al., 2016) indicated the beneficial effects of pioglitazone in reducing stroke and myocardial infarction in patients with insulin-resistant diabetes and cerebrovascular disease. As AF is associated with increased risk of stroke and cardiovascular events, this can partly be explained by the effects of pioglitazone in decreasing the AF burden (Stewart et al., 2002; Zhang Z. et al., 2017). In a meta-analysis of more than 3 million diabetic patients, our group recently reported that pioglitazone can protect against AF in diabetes (Zhang Z. et al., 2017).

Experimental studies indicate that pioglitazone can inhibit atrial remodeling by inhibiting the mitochondria-driven apoptosis (Xu et al., 2012), TGF-β1–Smad2/3 and TGF-β1–tumor necrosis factor receptor associated factor 6 (TRAF6)–TGF-β-associated kinase 1 (TAK1) (Gu et al., 2013), and NF-κB–TGF-β1–Toll/IL-1 receptor domain-containing adaptor inducing IFN-β (TRIF)–TRAF6 signaling pathway (Chen et al., 2015). Our group has previously used an alloxan-induced diabetes rabbit model for studying and characterizing the molecular mechanisms underlying diabetes-related AF development (Zhang X. et al., 2017; Yang et al., 2018). Using the same model here, we found that pioglitazone treatment prevented diabetes-induced adverse electrophysiological remodeling, which was reflected in the higher refractory period dispersion and conduction delay through the atria associated with interstitial fibrosis and higher AF incidence, which are substrates for AF.

The precise pathophysiological mechanisms implicating diabetes in AF development have not been completely identified, but oxidative stress and inflammation have been studied in recent years because they can induce structural and electrical remodeling in the atria (Tse et al., 2016a; Karam et al., 2017). Excessive ROS production is associated with oxidative damage of lipids, proteins, and DNA (Korantzopoulos et al., 2007; Tse et al., 2016b). As a major source of ROS in cardiomyocytes, mitochondria are also prone to oxidative damage and stress (Anderson et al., 2009; Shadel and Horvath, 2015).

Mitochondria are vital organelles in organisms that provide energy for cells through oxidative phosphorylation. The main physiological function of the mitochondria is ATP synthesis by oxidative phosphorylation. The mitochondria are also responsible for ROS generation and removal, Ca^2+^ homeostasis maintenance, metabolite synthesis and catabolism, and apoptosis regulation (Wang and Youle, 2009; Brand and Nicholls, 2011; Yuan et al., 2020). Many diseases, including diabetes and AF, are related to mitochondrial dysfunction, showing significant reduction in MMP and impaired respiratory function (Pieczenik and Neustadt, 2007; Wiersma et al., 2019).

Uncoupling proteins (UCPs), located in the inner mitochondrial membrane, play a major role in energy metabolism. Among them, UCP2 is ubiquitously expressed and is an important regulator of mitochondrial ROS production (Tian et al., 2018), exhibiting antioxidative stress activity under a variety of conditions (Liu et al., 2014). In the mitochondria isolated from UCP2 knockout (KO) mice, Cabrera *et al.* (2012) found that brief anoxia–reoxygenation resulted in impaired mitochondrial function, manifested by decreased state three respiration and RCR. Pioglitazone treatment increased UCP2 protein expression in heart tissue, along with mitochondrial membrane depolarization, and decreased maximal levels of superoxide species. These data indicate the protective role of pioglitazone in preventing oxidative stress damage by regulating UCP2 expression.

The burden of ROS is largely counteracted by SOD, catalase, and glutathione peroxidase (Finkel and Holbrook, 2000). PPAR-γ is a member of the nuclear hormone receptor superfamily of ligand-activated transcription factors, which has been verified as an important regulator of oxidative stress and inflammation (Liu and Li, 2012; Liu et al., 2013). It has been suggested that PPAR-γ critically regulates myocardial redox homeostasis by modulating Mn-SOD expression (Ding et al., 2007). In the present study, we found that pioglitazone increased serum PPAR-γ levels and SOD activity, reduced atrial mitochondrial ROS production, and suppressed the elevation in pro-oxidative and inflammatory markers in the serum (8-OHdG and hs-CRP) and LA (NF-κB) of diabetic rabbits. These findings support the hypothesis that oxidative stress and inflammation play significant roles in the development and perpetuation of AF, which pioglitazone treatment can reverse.

PGC-1α is a transcription coactivator of PPAR-γ that acts as a key regulator of mitochondrial biogenesis; it also scavenges cellular ROS production by inducing Mn-SOD expression (Fujisawa et al., 2009). PPAR-γ stimulation promotes mitochondrial biogenesis through the induction of PGC-1α (Miglio et al., 2009). PGC-1α stimulates the expression of NRF1, a transcriptional regulator that promotes the transcription of many nuclear-encoded mitochondrial genes, such as the oxidative phosphorylation genes and TFAM. TFAM is a direct regulator of mitochondrial DNA transcription/replication (Gao et al., 2010). In addition, PGC-1α also controls mitochondrial dynamics, a fusion cycle for forming networks, and fission to form smaller individual mitochondria that contribute to maintaining normal mitochondrial function and structure (Dabrowska et al., 2015). Increased ROS triggers imbalanced mitochondrial dynamics, and altered mitochondrial dynamics have also been associated with increased mitochondrial ROS production (Shenouda et al., 2011). Consistent with our previous findings (Zhang X. et al., 2017), we report here that the diabetic rabbits exhibited decreased protein levels of PGC-1α, NRF1, and TFAM. Moreover, diabetes induced atrial mitochondrial swelling, mitochondrial respiratory dysfunction, and MMP collapse; however, pioglitazone treatment ameliorated these changes.

Mitochondria are dynamic organelles; depending on fusion and fission events, mitochondrial dynamics are critical for regulating morphology, number, function, and subcellular distribution (Meyer et al., 2017). Mitochondrial fusion and fission require the regulation of key proteins, including the mitochondrial fusion proteins such as Opa1, Mfn1, and Mfn2 and the mitochondrial fission proteins such as Drp1 and Fis1, which are regulated by PGC-1α (Lopez-Lluch, 2017). Abnormalities in these factors can lead to the accumulation of disabled mitochondria that increase ROS production (Lopez-Lluch, 2017). In the present study, we found that the diabetic rabbits had decreased expression of the mitochondrial fusion (Opa1 and Mfn1)- and fission (Drp1)-related proteins, while pioglitazone promoted their expression. In HL-1 cells, PGC-1α siRNA transfection blunted the effects of pioglitazone on Mn-SOD protein expression in H_2_O_2_-treated cells. In keeping with the *in vivo* findings, pioglitazone increased the serum SOD activity and reduced the atrial mitochondrial ROS production of diabetic rabbits, suggesting that it produces cardiovascular benefits by promoting ROS removal through the PPAR-γ/PGC-1α signaling pathway.

## Limitations

Several limitations of our study should be noted. First, we used pioglitazone to evaluate the effects of the PPAR-γ/PGC-1α signaling pathway on mitochondrial oxidative stress in diabetic rabbits. However, this drug does not have 100% specificity for these proteins, and nonspecific or off-target effects could not be ruled out. Second, the electron microscopy measurements were not quantified. Third, we did not examine the expression of PPAR-γ and the classic PPAR-γ target genes in cardiac tissue and HL-1 cells. Further studies are needed to detail all of the mechanisms by which the PPAR-γ/PGC-1α signaling pathway contributes to atrial adaptation of diabetes-induced mitochondrial oxidative stress. For example, PPAR-γ antagonists could be used, and PPAR-γ siRNA experiments could be conducted.

## Conclusion

The PPAR-γ agonist pioglitazone reduces mitochondrial ROS production, inhibits oxidative stress and inflammation, and improves mitochondrial biogenesis, dynamics, and function through the PPAR-γ/PGC-1α signaling pathway. Together, these findings explain the atrial reverse remodeling and the reduction in the incidence of inducible AF in our rabbit model of diabetes mellitus.

## Data Availability

The raw data supporting the conclusions of this article will be made available by the authors, without undue reservation, to any qualified researcher.
